# Phase 1 trial of lavage cytology collected with endoscopic ultrasound-guided fine-needle aspiration for preoperative pancreatic cancer

**DOI:** 10.1055/a-2781-5703

**Published:** 2026-02-13

**Authors:** Nozomi Okuno, Kazuo Hara, Shin Haba, Takamichi Kuwahara, Shimpei Matsumoto, Hiroki Koda, Keigo Oshiro, Tomoki Ogata, Yuki Uba, Tomoyuki Tanaka, Yuma Yamazaki, Saki Yamazoe

**Affiliations:** 1 538357 Gastroenterology, Aichi Cancer Center, Nagoya, Japan

**Keywords:** Endoscopic ultrasonography, Intervention EUS, Pancreas, Fine-needle aspiration/biopsy

## Abstract

**Background and study aims:**

Intraoperative peritoneal lavage cytology (CY) influences prognosis in pancreatic cancer, and positive findings may lead to aborted surgery. Staging laparoscopy is traditionally used when no distant metastases are evident. We developed a novel method to assess CY using endoscopic ultrasound (EUS) and conducted a Phase 1 trial to evaluate its safety.

**Patients and methods:**

This non-randomized, prospective Phase I1trial was conducted in three stages with safety monitoring after each stage. The study was approved by the institutional review board (2023–0-239) and registered (UMIN000052528). Nine patients with pancreatic ductal adenocarcinoma who were considering surgery were enrolled between September 2023 and August 2024. A 3F sheath was inserted into the upper abdomen under endoscopic and fluoroscopic guidance, followed by injection of 200 to 300 mL of saline. After postural adjustments and abdominal massage, transrectal EUS-fine-needle aspiration was used to aspirate pelvic fluid.

**Results:**

Median age was 68 years (range, 36–80); 77.8% were male. Resectability status: resectable/borderline/unresectable (considering conversion): 4/2/3. The procedure was successful in all cases, with a median aspirated volume of 32 mL (range, 10–125). No adverse events occurred, and all patients were discharged the next day.

**Conclusions:**

This novel EUS-guided lavage cytology method was safe and feasible. A Phase 2 trial is planned.

## Introduction

In pancreatic cancer, a positive result in intraoperative peritoneal lavage cytology is classified as M1 and corresponds to stage IV 1 2 . Several studies have reported that even if surgical resection is performed, patients with positive lavage cytology have significantly shorter overall survival, making it a critical prognostic factor 3 . Although preoperative imaging may not reveal peritoneal dissemination, peritoneal lavage cytology performed during staging laparoscopy or surgery may still yield positive results 4 5 . Traditionally, staging laparoscopy has been performed for cases without detectable metastases on other imaging modalities. However, it requires general anesthesia, which poses physical and financial burdens for the patient. Given the rapid progression of pancreatic cancer, early initiation of treatment is essential, and it is meaningful to be able to avoid surgical preparations.

In this study, we devised a novel, minimally invasive technique for peritoneal lavage cytology, in which saline is percutaneously infused into the peritoneal cavity and ascitic fluid is collected transrectally under endoscopic ultrasound (EUS) guidance.

Although previous reports have described percutaneous infusion and percutaneous retrieval of saline for cytological analysis 6 , to our knowledge, no studies have utilized EUS for peritoneal lavage cytology as described in our approach. Therefore, we conducted this Phase 1 trial to evaluate the safety of this novel method: EUS-guided lavage cytology.

## Patients and methods

### Study design

This study was a single-arm, prospective, single-center Phase 1 trial conducted in three stages. If no more than one case of unacceptable procedure-related adverse events (AEs) had occurred among the first three patients in the first stage, the trial was to proceed to the second stage. After the procedures had been completed in three additional patients in the second stage (a total of six patients), a 30-day enrollment suspension period was implemented to confirm safety. If no more than one unacceptable AE had occurred among the three patients in the second stage, the trial proceeded to the third stage involving three more patients. The trial was terminated if three cases of unacceptable procedure-related AEs occurred, including those in the first stage and additional enrollments. The hospital ethics review board approved the study (2023–0-239). This trial was registered in the UMIN clinical trials registry (UMIN000052528).

Inclusion criteria were the following: 1) age ≥ 20 years; 2) provision of written informed consent by the patient; 3) pancreatic cancer diagnosable by imaging modalities; and 4 EUS-fine-needle aspiration (EUS-FNA) deemed feasible and safe to perform. Exclusion criteria were the following: 1) presence of synchronous cancer currently under treatment; 2) ascites or disseminated peritoneal nodules detected on imaging; 3) known allergy to lidocaine (Xylocaine); and 4) deemed inappropriate for study participation by the attending physician.

For the primary endpoint, frequency of all AEs arising from the study procedure was calculated, and their severity was assessed. Evaluation of AEs was conducted in accordance with CTCAE version 5.0 7 , whereas severity was assessed using “A Lexicon for Endoscopic Adverse Events: Report of an ASGE Workshop” 8 . AEs clearly unrelated to the study procedure were excluded from the evaluation.

Unacceptable AEs included the following events, occurring within 30 days after procedures: 1) fever (≥ 38 °C) lasting > 7 days; 2) inability to resume oral intake > 7 days; 3) prolonged hospitalization > 14 days; 4) requiring additional intervention due to AEs from this procedure; and 5) AEs classified as “severe” in the severity assessment.

As a secondary endpoint, the positive rate of peritoneal lavage cytology was evaluated among all enrolled patients.

### Study procedures

Following confirmation of avascularity with Doppler, a fine-needle aspiration (FNA) needle is used to puncture the target area, and fluid is aspirated as completely as possible under real-time EUS guidance.Video 1

**Fig. 1:**
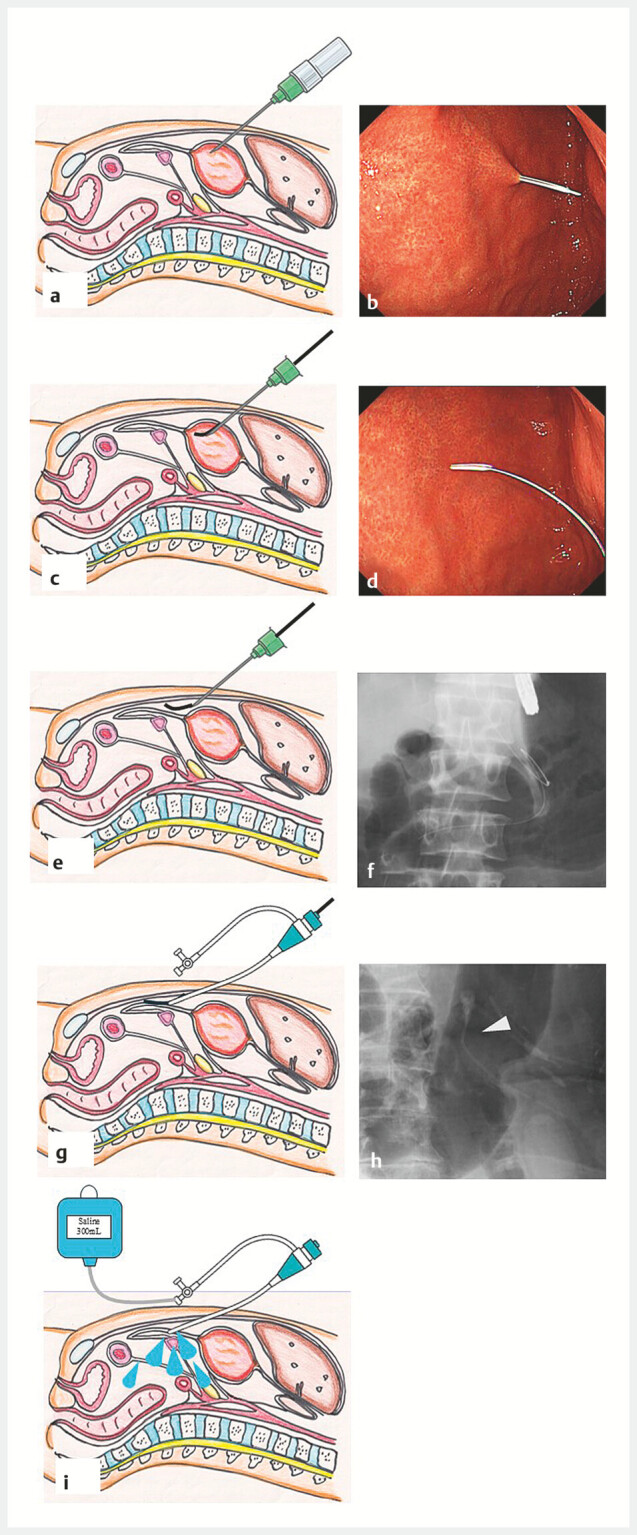
Study procedures. **a** Schematic illustration showing puncture of the anterior gastric wall using a Surflo. **b** Endoscopic image showing puncture of the anterior gastric wall with a Surflo. **c** Following placement of the outer cannula, a guidewire is advanced. **d** Endoscopic image showing advancement of the guidewire. **e** The outer cannula is slightly withdrawn, and the guidewire is advanced caudally into the peritoneal cavity. **f** Under fluoroscopic guidance, the guidewire is positioned in the caudal direction within the abdominal cavity. **g** A 3F sheath is inserted into the peritoneal cavity over the guidewire. **h** The white arrowhead indicates the sheath. **i** Saline is injected into the peritoneal cavity through the sheath.

Bowel cleansing was performed in accordance with the standard preparation for colonoscopy, followed by routine endoscopic preparation ( Fig. 1 ). All patients were under conscious sedation with intravenous medication during the procedure and received intravenous prophylactic antibiotics.

First, an upper endoscope was inserted into the stomach. The anterior gastric wall was punctured using an 18G Surflo (18G Surflo 2 1/2", TERUMO, Japan), and the outer cannula was placed. A guidewire was then inserted into the abdominal cavity, and after confirming its position via endoscopy and fluoroscopy, the device was exchanged for a 3F sheath (Super Sheath 3Fr × 11 cm, Medikit, Japan).

A total of 200–300 mL of saline was injected into the abdominal cavity. Postural changes, abdominal massage, and head-up positioning were performed. Transrectal EUS-FNA (22–19 Gauge, with or without side holes; EZ Shot 3 Plus, Olympus, Japan) was performed to puncture and aspirate the ascitic fluid accumulated in the pelvic cavity, retrieving as much fluid as possible ( Video 1 ).

The protocol procedure was considered successful if at least 10 mL of ascitic fluid was retrieved.

## Results

### Patient characteristics

**Fig. 2:**
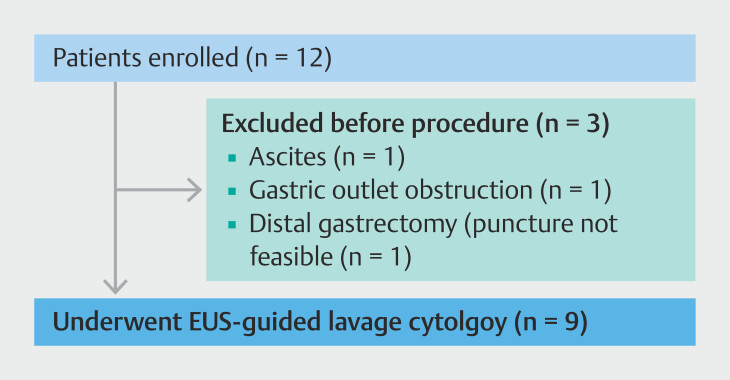
Flowchart of patient enrollment and exclusion.

During the period from September 2023 to August 2024, 12 patients were enrolled. Of these, three did not undergo the study procedure: one patient developed new-onset ascites, another developed gastric outlet obstruction prior to endoscopy, and in one patient with a history of distal gastrectomy, upper endoscopy was performed but the procedure was not initiated due to inability to ensure a safe puncture route. Therefore, the study procedure was performed in nine patients ( Fig. 2 ).

Patient characteristics are listed in Table 1 . Median age was 68 years (range, 36–80 years) and 77.8% were men (7 men and 2 women). The baseline resectability classification was as follows: resectable/borderline resectable/unresectable with metastasis considered for conversion surgery: 4/2/3. Seven of the nine patients (77.8%) had received chemotherapy prior to the procedure.

**Table:** **Table 1** Patient characteristics.

Age, years; median [range]	68 {36–80]
Sex (male/female)	7/2
Performance status (0/1)	8/1
Antiplatelet/anticoagulant use	0
Tumor location (head/body and tail)	6/3
Baseline resectability status (R/BR/UR-LA/UR-M*)	4/2/0/3
Prior chemotherapy(yes/no)	7/2
BR, borderline resectable; R, resectable; UR-LA, unresectable locally advanced; UR-M, unresectable with distant metastasis. *Considering conversion surgery.	

### Procedure outcomes

Procedure outcomes are summarized in Table 2 . Technical success was achieved in all nine cases (100%). Median volume of saline injected was 300 mL (range: 250–300). Median number of FNA passes was three (range: 1–4). Median volume of ascitic fluid aspirated was 32 mL (range: 10–125) and median procedure time was 52 minutes (range: 20–90). Procedure time was defined as duration from insertion of the upper endoscope to completion of lower EUS-FNA.

**Table:** **Table 2** Procedure outcomes.

Technical success rate, % (n/N)	100 (9/9)
Volume of saline injected, median (mL) [range]	300 [250–300]
Number of FNA passes, median [range]	3 [1–4]
Needle gauge, 19/22	1/8
Needle side hole, with/without	2/7
Volume of ascitic fluid aspirated, median (mL) [range]	32 [10–125]
Time required for procedure*, median (min) [range]	52 [20–90]
Technical success rate, % (n/N)	100 (9/9)
FNA, fine-needle aspiration. *Insertion of the upper endoscope to completion of lower EUS-FNA	

### Safety outcomes

Safety outcomes are summarized in Table 3 . No procedure-related AEs were observed in any of the nine patients (0/9, 0%). Specifically, there were no cases of fever, peritonitis, bleeding, or infection. There was no procedure-related mortality, and no patients required additional interventions.

**Table:** **Table 3** Safety outcomes.

Adverse events, % (n/N)	0 (0/9)
Fever	0
Peritonitis	0
Bleeding	0
Infection	0
Procedure-related mortality	0
Additional intervention required	0
Time to resume oral intake, median (days) [range]	1 [1–1]
Post-procedure stay, median (days) [range]	1 [1–1]
Prolonged hospitalization	0

All patients resumed oral intake on the day following the procedure and were subsequently discharged.

### Cytology findings and clinical course

Cytology findings and clinical courses are summarized in Table 4 . EUS-guided peritoneal lavage cytology was negative in all nine patients. Of these, six patients proceeded to surgical resection, with time to surgery ranging from 12 to 301 days. Intraoperative peritoneal lavage cytology was performed in all surgical cases and was negative in every instance. Two patients did not undergo surgery due to newly identified liver metastasis (n = 1) or continuation of chemotherapy for metastatic disease (n = 1). Among the six surgical cases, three patients experienced recurrence, involving the lymph nodes, lung, or liver. Notably, no cases of peritoneal dissemination were detected.

**Table:** **Table 4** Cytological findings and clinical course.

**No**	**Baseline resectability**	**EUS-guided lavage cytology**	**Time to surgery (days)**	**Reason for not undergoing surgery**	**Intraoperative peritoneal lavage cytology**
1	R	Negative	12		Negative
2	R	Negative	13		Negative
3	R	Negative	93		Negative
4	R	Negative	-	New liver metastasis	-
5	BR-A	Negative	26		Negative
6	BR-PV	Negative	27		Negative
7	UR-M (LN)	Negative	301		Negative
8	UR-M (HEP)	Negative	59		Negative
9	UR-M (LN)	Negative	-	Continued chemotherapy	-
BR, borderline resectable; HEP, hepatic; LN, lymph node; PUL, pulmonary; R, resectable; UR-LA, unresectable locally advanced; UR-M, unresectable with distant metastasis.					

## Discussion

We conducted a phase I trial using a novel EUS-guided technique to evaluate peritoneal cytology in patients with pancreatic cancer to assess safety. The procedure was successfully performed in all cases and no procedure-related AEs were observed. In systematic review 9 , the cumulative complication rate of EUS-FNA was reported to be 0.98% among 10,941 patients enrolled across 51 studies. However, subgroup analysis based on the target organ revealed that the highest complication rate was observed in patients who underwent EUS-FNA for ascites (3.53%), followed by patients undergoing EUS-FNA for hepatic (2.33%) and perirectal (2.07%) lesions. Of the 85 patients who underwent EUS-FNA for ascites, complications developed in three, including two with transient fever and one with bacterial peritonitis, which resolved after antibiotic therapy. A retrospective analysis of our prior experience with 13 patients who underwent transcolonic EUS-FNA for extrinsic colonic lesions revealed no AEs 10 . However, as noted in the systematic review, particular attention should be paid to infection prevention when performing transcolonic EUS-FNA for fluid collection. One area for improvement identified in our results is reduction in number of FNA needle passes. In the early cases, a conventional FNA needle was used; however, once fluid was aspirated, the tip of the needle often became occluded by adjacent small bowel, preventing further aspiration. Implementation of a side-hole FNA needle could potentially reduce risk of this complication and decrease the number of needle passes, which should be considered as a point of refinement in future procedures.

In addition to safety, we investigated characteristics of our new method in comparison with previously reported percutaneous approaches. Our new method involves injection of saline from the upper abdomen, followed by abdominal massage and head-up positioning. Fluid is then collected from the lower abdomen using EUS. We hypothesized that this approach would enhance detection of peripancreatic dissemination.

Pak et al. 6 and Sugawara et al. 11 have reported evaluation of peritoneal cytology in pancreatic cancer using percutaneous approaches. The most notable difference between these previous reports and our novel method is that the routes for saline injection and fluid collection are separate in our approach. Moreover, our technique incorporates upper abdominal saline injection, abdominal massage, and head-up positioning, which may facilitate movement of free cancer cells into dependent regions, thereby improving their detectability.

Pak et al. 6 inserted a catheter into the left upper abdomen in 22 patients with pancreatic cancer, infused saline, and retrieved the fluid from the same site. A median of 800 mL of saline was infused and a median of 60 mL was successfully recovered. The authors reported the procedure to be both safe and feasible. Similarly, Sugawara et al. 9 conducted a retrospective study evaluating utility of percutaneous abdominal lavage cytology in 44 patients with resectable pancreatic cancer. In this study, two different access routes were utilized. Under compute tomography guidance, a drainage catheter was inserted into the pouch of Douglas via either the right abdomen or the suprapubic route, and lavage was performed through the same site. In addition, a significant advantage of our technique is that fluid retrieval is conducted under direct EUS visualization, as opposed to the blind aspiration used in previous percutaneous approaches. This enables precise targeting. Nonetheless, the percutaneous approach offers the advantage of maintaining sterility and lower risk of infection. Although no AEs, including infection, occurred in this trial, preventive measures to further reduce the risk of infection should still be considered, as noted earlier.

## Conclusions

In conclusion, our novel EUS-guided lavage cytology technique for patients with pancreatic cancer was shown to be safe and technically feasible. This approach combines upper abdominal saline injection with EUS-guided aspiration from the lower abdomen, offering real-time visualization, targeted sampling, and the potential for improved detection of peripancreatic dissemination. Although this phase I study focused primarily on safety, further refinement of the techniques—such as optimizing needle design and minimizing infection risk—is warranted. A prospective Phase 2 trial should be conducted to evaluate its diagnostic performance and clinical utility, particularly in detecting subclinical peritoneal dissemination and informing treatment strategies.
